# (Glycol-κ^2^ *O*,*O*′)nitros­yl(η^5^-penta­methyl­cyclo­penta­dien­yl)ruthenium(II) bis­(tri­fluoro­methane­sulfonate)

**DOI:** 10.1107/S1600536807067426

**Published:** 2008-01-04

**Authors:** Semeret Munie, Anna Larsen, Milan Gembicky

**Affiliations:** a Department of Chemistry, CNS 359, Ithaca College, Ithaca, NY 14850, USA; b Department of Chemistry, State University of New York at Buffalo, 732 NSC Complex, Buffalo, NY 14260, USA

## Abstract

The title compound, [Ru(C_10_H_15_)(NO)(HOCH_2_CH_2_OH)](CF_3_SO_3_)_2_, possesses a three-legged piano-stool geometry around the Ru atom, with an average Ru—O distance of 2.120 (6) Å and an Ru—N—O angle of 159.45 (14)°. The ethyl­eneglycol ligand forms a non-planar metallacyclic ring by chelating the Ru atom *via* the O atoms. The O⋯O distances of 2.554 (2) and 2.568 (2) Å are indicative of hydrogen bonding between coordinated ethyl­eneglycol and outer-sphere trifluoro­methane­sulfonate fragments. The crystal packing is stabilized by ionic forces and several CH_3_⋯·F (2.585 and 2.640 Å) and CH_3_⋯O inter­actions (2.391, 2.678, 2.694 and 2.699 Å) between the penta­methyl­cyclo­penta­dienyl ligand and trifluoro­methane­sulfonate anion. There is noticeable short inter­molecular contact [2.9039 (16) Å], between an O atom of the SO_3_ group and a C atom of the penta­methyl­cyclo­penta­dienyl ligand.

## Related literature

For closely related ruthenium diol- and alkyl­oxy-chelated structures, see: Hubbard & McVicar (1992 ▶); Yang *et al.* (1995 ▶, 1997 ▶). For chemicaly related complexes, see: Burns & Hubbard (1994 ▶); Pearsal *et al.* (2007 ▶); Svetlanova-Larsen *et al.* (1996 ▶).
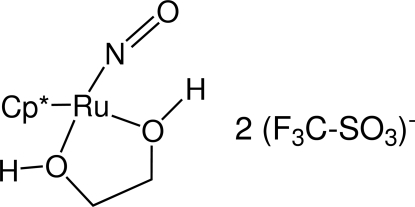


## Experimental

### 

#### Crystal data

[Ru(C_10_H_15_)(NO)(C_2_H_6_O_2_)](CF_3_O_3_S)_2_
*M* *_r_* = 626.51
Monoclinic, 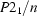

*a* = 8.5593 (2) Å
*b* = 30.5443 (7) Å
*c* = 8.8608 (2) Å
β = 91.295 (1)°
*V* = 2315.96 (9) Å^3^
*Z* = 4
Mo *K*α radiation
μ = 0.95 mm^−1^
*T* = 90 (1) K
0.20 × 0.20 × 0.04 mm

#### Data collection

Bruker SMART APEX2 diffractometer
Absorption correction: multi-scan (*SADABS*; Bruker, 2004 ▶) *T*_min_ = 0.833, *T*_max_ = 0.963
30259 measured reflections
5102 independent reflections
4513 reflections with *I* > 2σ(*I*)
*R*_int_ = 0.027

#### Refinement

*R*[*F* ^2^ > 2σ(*F* ^2^)] = 0.021
*wR*(*F* ^2^) = 0.049
*S* = 1.04
5102 reflections
311 parameters
H atoms treated by a mixture of independent and constrained refinement
Δρ_max_ = 0.52 e Å^−3^
Δρ_min_ = −0.37 e Å^−3^

### 

Data collection: *APEX2* (Bruker, 2004 ▶); cell refinement: *APEX2*; data reduction: *SAINT* (Bruker, 2004 ▶); program(s) used to solve structure: *SHELXS97* (Sheldrick, 1997 ▶); program(s) used to refine structure: *SHELXL97* (Sheldrick, 1997 ▶); molecular graphics: *SHELXTL* (Sheldrick, 2000 ▶); software used to prepare material for publication: *publCIF* (Westrip, 2008 ▶).

## Supplementary Material

Crystal structure: contains datablocks I, global. DOI: 10.1107/S1600536807067426/bg2160sup1.cif

Structure factors: contains datablocks I. DOI: 10.1107/S1600536807067426/bg2160Isup2.hkl

Additional supplementary materials:  crystallographic information; 3D view; checkCIF report

## Figures and Tables

**Table 1:** Hydrogen-bond geometry (Å, °)

*D*—H⋯*A*	*D*—H	H⋯*A*	*D*⋯*A*	*D*—H⋯*A*
O2—H2⋯O5	0.80 (3)	1.76 (3)	2.568 (2)	176 (3)
O3—H3⋯O7	0.80 (3)	1.76 (3)	2.554 (2)	169 (3)

**Fig. 1.**
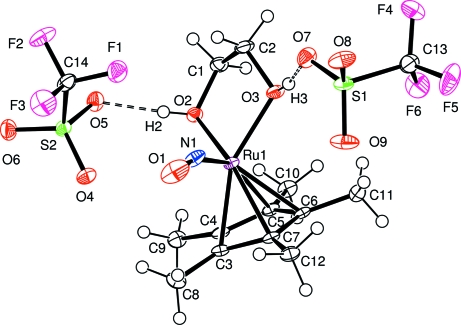
, Molecular structure of the title compound. Displacement ellipsoids are drawn at the 50% probability level. Hydrogen bonds shown in broken lines.

**Fig. 2.**
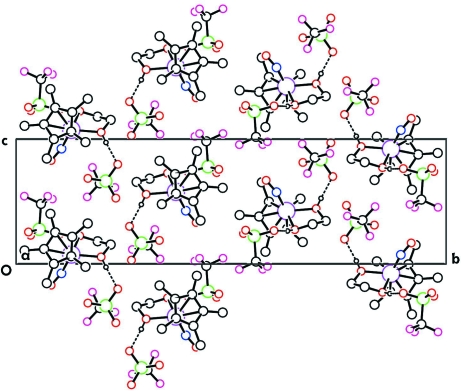
A packing diagram of the title compound, showing the columns of Ru-complex and trifluoromethanesulfonate anion (coming out of the plane) along the a-axis direction. Only hydrogen atoms involved in H-bonding are present. Hydrogen bonds are shown as dashed lines.

## supplementary crystallographic information

### Comment

The electrophilic ruthenium center in trifluoromethanesulfonate complexes with a [(C_10_H_15_)Ru(NO)] core is reactive towards small molecular nucleophiles, such as water (Svetlanova-Larsen *et al.*, 1996), unsaturated hydrocarbons (Burns & Hubbard, 1994), and alcohols, and results in their binding and activation. For primary and secondary alcohols, after initial coordination, the reaction leads to alcohol oxidation and reduction of the ruthenium center to the Ru=Ru dimer, (Pearsal *et al.*, 2007).In the case of ethylene glycol, however, the coordination product is stabilized by a chelate ring formation and can be isolated and characterized.

### Experimental

The compound was obtained as a product of reaction between Cp*Ru(NO)(SO_3_CF_3_)_2_ and 3 equivalents of ethylene glycol in chloroform solution (Cp* = C_5_Me_5_). The single crystals for the X-ray diffraction studies were grown by solvent diffusion from a dichloromethane/hexane mixture at ambient temperature under inert atmosphere (Pearsal *et al.*, 2007).

All synthetic procedures were carried out in inert atmosphere. 10 µ*L* of HO—CH_2_CH_2_—OH (0.213 mmol, 3 equiv) was added to a stirred solution of 40 mg of Cp*Ru(NO)(SO_3_CF_3_)_2_ (0.071 mmol) in 10 ml of CHCl_3_. A red precipitate formed within 5 min and the initially purple solution became almost colorless. The supernatant was decanted and the precipitate was recrystallized from a CH_2_Cl_2_/hexane mixture at -40 °C yielding 30 mg (0.050 mmol, 70%) of analytically pure complex[(C_10_H_15_)Ru(NO)(OH—CH_2_—CH_2_—OH)]^2+^ 2[SO_3_CF_3_]^-^. X-ray quality crystals were grown by slow diffusion of hexane into dichloromethane solution. The CHCl_3_ solvent used in this reaction should be completely ethanol-free, otherwise isolation of the crystalline product becomes difficult due to the reaction between Cp*Ru(NO)(SO_3_CF_3_) and ethanol used routinely for stabilization of commercially available chloroform. The compound was characterized by 1H, 19 F, 13 C NMR, and by IR spectroscopy. 1H NMR(CH_2_Cl_2_): δ 1.93 (*s*) (15*H*, Cp*); δ 1.88 (*s*) (15*H*, Cp* minor amounts of starting material existing in equilibrium with product); δ 4.23 (broad) (2*H*, HO-CHH_a_—CHH_a_—OH); δ 3.35 (broad) (2*H*, HO—CH_b_H—CH_b_H—OH); δ 11.13 (2*H*, 2OH); 13 C NMR (CH_2_Cl_2_): δ 119.8 (q, SO_3_CF_3_, JC—F = 318.4 Hz); δ 113.9 (C_5_Me_5_); δ 67.8 (HO—CH_2_—CH_2_—OH), δ 9.6 (C_5_Me_5_); 19F{1H} NMR (CH_2_Cl_2_): δ -78.6; IR (nujol) vNO 1820 cm-1 (*versus*); mp 131 C; Anal. Calcd for C_14_H_21_NO_9_RuS_2_F_6_ (626): C, 26.80; H, 3.40; N, 2.20; Found: C, 26.82; H, 3.43; N, 2.18.

### Refinement

All non-hydrogen atoms were refined anisotropically. Positions of hydrogen atoms were found from difference Fourier maps, but placed in calculated position and refined in the riding approximation. CH_3_ H atoms were treated as part of idealized methyl group with torsion angles from electron density. Displacement factors were assigned as *U*_iso_=1.5*U*_eq_ (for CH~3~) and *U*_iso_=1.2*U*_eq_ for the CH_2_. Hydroxyl hydrogen atoms were freely refined.

### Crystal data

**Table:**

[Ru(C_10_H_15_)(NO)(C_2_H_6_O_2_)](CF_3_O_3_S)_2_	*F*_000_ = 1256
*M**_r_* = 626.51	*D*_x_ = 1.797 Mg m^−^^3^
Monoclinic, *P*2_1_/*n*	Mo *K*α radiation λ = 0.71073 Å
Hall symbol: -P2yn	Cell parameters from 6176 reflections
*a* = 8.5593 (2) Å	θ = 2.4–27.1º
*b* = 30.5443 (7) Å	µ = 0.95 mm^−^^1^
*c* = 8.8608 (2) Å	*T* = 90 (1) K
β = 91.295 (1)º	Plate, red
*V* = 2315.96 (9) Å^3^	0.20 × 0.20 × 0.04 mm
*Z* = 4	

### Data collection

**Table:**

Bruker SMART APEX2 diffractometer	5102 independent reflections
Radiation source: rotating anode	4513 reflections with *I* > 2σ(*I*)
Monochromator: graphite	*R*_int_ = 0.027
Detector resolution: 8.33 pixels mm^-1^	θ_max_ = 27.1º
*T* = 90(1) K	θ_min_ = 1.3º
ω scans	*h* = −10→10
Absorption correction: multi-scan(SADABS; Bruker, 2004)	*k* = −39→39
*T*_min_ = 0.833, *T*_max_ = 0.963	*l* = −11→11
30259 measured reflections	

### Refinement

**Table:**

Refinement on *F*^2^	Secondary atom site location: difference Fourier map
Least-squares matrix: full	Hydrogen site location: difference Fourier map
*R*[*F*^2^ > 2σ(*F*^2^)] = 0.021	H atoms treated by a mixture of independent and constrained refinement
*wR*(*F*^2^) = 0.049	*w* = 1/[σ^2^(*F*_o_^2^) + (0.0207*P*)^2^ + 1.8018*P*] where *P* = (*F*_o_^2^ + 2*F*_c_^2^)/3
*S* = 1.04	(Δ/σ)_max_ = 0.003
5102 reflections	Δρ_max_ = 0.52 e Å^−^^3^
311 parameters	Δρ_min_ = −0.37 e Å^−^^3^
Primary atom site location: structure-invariant direct methods	Extinction correction: none

### Special details

**Table:**

Experimental. X-ray diffraction data on the title compound were collected at 90 (1) K using a Bruker *SMART APEX2* CCD diffractometer installed at a Rigaku rotating anode source (Mo K\a radiation, λ =0.71073 Å), and equipped with an Oxford Cryosystems nitrogen gas-flow apparatus. Data collection was performed with four runs at φ = 0.00 °, at φ = 90.00 °, at φ = 180 ° and at φ = 270 ° (600 frames each). Frame width = 0.30 ° in ω. Data were merged, corrected for decay, and treated with multi-scan absorption corrections, Bruker (2004).
Geometry. All e.s.d.'s (except the e.s.d. in the dihedral angle between two l.s. planes) are estimated using the full covariance matrix. The cell e.s.d.'s are taken into account individually in the estimation of e.s.d.'s in distances, angles and torsion angles; correlations between e.s.d.'s in cell parameters are only used when they are defined by crystal symmetry. An approximate (isotropic) treatment of cell e.s.d.'s is used for estimating e.s.d.'s involving l.s. planes.
Refinement. Refinement of *F*^2^ against ALL reflections. The weighted *R*-factor *wR* and goodness of fit *S* are based on *F*^2^, conventional *R*-factors *R* are based on *F*, with *F* set to zero for negative *F*^2^. The threshold expression of *F*^2^ > σ(*F*^2^) is used only for calculating *R*-factors(gt) *etc*. and is not relevant to the choice of reflections for refinement. *R*-factors based on *F*^2^ are statistically about twice as large as those based on *F*, and *R*- factors based on ALL data will be even larger.

### Fractional atomic coordinates and isotropic or equivalent isotropic displacement parameters (Å^2^)

**Table:**

	*x*	*y*	*z*	*U*_iso_*/*U*_eq_	
Ru1	0.363831 (15)	0.628334 (4)	0.422226 (16)	0.01184 (4)	
S1	0.79684 (5)	0.553701 (14)	0.22295 (5)	0.01778 (10)	
S2	0.38389 (5)	0.708576 (14)	0.83367 (5)	0.01840 (10)	
F1	0.64453 (14)	0.67755 (4)	0.72541 (14)	0.0311 (3)	
F2	0.67642 (15)	0.72972 (4)	0.88629 (16)	0.0364 (3)	
F3	0.61548 (16)	0.66532 (4)	0.96169 (15)	0.0373 (3)	
F4	0.92738 (16)	0.58431 (5)	−0.01957 (15)	0.0393 (3)	
F5	0.83784 (17)	0.51903 (5)	−0.04318 (17)	0.0461 (4)	
F6	0.67972 (15)	0.57350 (4)	−0.04329 (14)	0.0350 (3)	
O1	0.51407 (19)	0.58134 (5)	0.66923 (18)	0.0359 (4)	
O2	0.37028 (15)	0.69766 (4)	0.44193 (15)	0.0160 (3)	
H2	0.372 (3)	0.7089 (8)	0.524 (3)	0.038 (7)*	
O3	0.54090 (15)	0.64795 (4)	0.27409 (15)	0.0183 (3)	
H3	0.611 (3)	0.6309 (8)	0.263 (3)	0.035 (7)*	
O4	0.30874 (16)	0.66687 (4)	0.81175 (16)	0.0237 (3)	
O5	0.38100 (16)	0.73629 (4)	0.69937 (15)	0.0219 (3)	
O6	0.35026 (17)	0.73178 (4)	0.97014 (16)	0.0265 (3)	
O7	0.78423 (16)	0.59968 (4)	0.26746 (16)	0.0252 (3)	
O8	0.94083 (15)	0.53366 (4)	0.27126 (18)	0.0268 (3)	
O9	0.65658 (16)	0.52870 (5)	0.24344 (18)	0.0311 (3)	
N1	0.47625 (18)	0.60542 (5)	0.57437 (19)	0.0210 (3)	
C1	0.4776 (2)	0.71981 (6)	0.3435 (2)	0.0193 (4)	
H1A	0.5153	0.7473	0.3911	0.023*	
H1B	0.4246	0.7271	0.2463	0.023*	
C2	0.6118 (2)	0.68950 (6)	0.3177 (2)	0.0207 (4)	
H2A	0.6785	0.7007	0.2365	0.025*	
H2B	0.6765	0.6861	0.4111	0.025*	
C3	0.16359 (19)	0.58780 (5)	0.4961 (2)	0.0152 (3)	
C4	0.10739 (19)	0.62820 (6)	0.4356 (2)	0.0155 (3)	
C5	0.15106 (19)	0.63035 (6)	0.2792 (2)	0.0148 (3)	
C6	0.23367 (19)	0.59090 (5)	0.2443 (2)	0.0144 (3)	
C7	0.24397 (19)	0.56501 (5)	0.3780 (2)	0.0139 (3)	
C8	0.1359 (2)	0.57127 (6)	0.6516 (2)	0.0216 (4)	
H8A	0.1364	0.5959	0.7225	0.032*	
H8B	0.2187	0.5506	0.6808	0.032*	
H8C	0.0344	0.5565	0.6537	0.032*	
C9	0.0145 (2)	0.66126 (6)	0.5195 (2)	0.0203 (4)	
H9A	−0.0942	0.6514	0.5248	0.030*	
H9B	0.0183	0.6894	0.4668	0.030*	
H9C	0.0586	0.6646	0.6219	0.030*	
C10	0.1076 (2)	0.66649 (6)	0.1722 (2)	0.0194 (4)	
H10A	0.1765	0.6657	0.0854	0.029*	
H10B	0.1190	0.6947	0.2238	0.029*	
H10C	−0.0010	0.6627	0.1373	0.029*	
C11	0.3010 (2)	0.57971 (6)	0.0955 (2)	0.0188 (4)	
H11A	0.3937	0.5613	0.1113	0.028*	
H11B	0.3304	0.6067	0.0433	0.028*	
H11C	0.2231	0.5638	0.0341	0.028*	
C12	0.3081 (2)	0.51965 (5)	0.3901 (2)	0.0177 (4)	
H12A	0.2255	0.4985	0.3648	0.027*	
H12B	0.3467	0.5144	0.4935	0.027*	
H12C	0.3942	0.5162	0.3200	0.027*	
C13	0.8112 (2)	0.55776 (6)	0.0190 (2)	0.0236 (4)	
C14	0.5906 (2)	0.69451 (6)	0.8531 (2)	0.0226 (4)	

### Atomic displacement parameters (Å^2^)

**Table:**

	*U*^11^	*U*^22^	*U*^33^	*U*^12^	*U*^13^	*U*^23^
Ru1	0.01021 (7)	0.00918 (6)	0.01600 (7)	−0.00059 (5)	−0.00226 (5)	0.00032 (5)
S1	0.0120 (2)	0.0172 (2)	0.0240 (2)	−0.00174 (16)	−0.00207 (17)	0.00336 (17)
S2	0.0227 (2)	0.0141 (2)	0.0184 (2)	−0.00311 (17)	0.00084 (18)	−0.00146 (17)
F1	0.0254 (6)	0.0365 (7)	0.0316 (7)	0.0005 (5)	0.0013 (5)	−0.0093 (5)
F2	0.0304 (7)	0.0261 (6)	0.0523 (9)	−0.0110 (5)	−0.0083 (6)	−0.0085 (6)
F3	0.0422 (8)	0.0333 (7)	0.0360 (7)	0.0061 (6)	−0.0080 (6)	0.0122 (6)
F4	0.0344 (7)	0.0521 (8)	0.0320 (7)	−0.0134 (6)	0.0114 (6)	0.0015 (6)
F5	0.0525 (9)	0.0368 (8)	0.0487 (9)	0.0094 (7)	−0.0059 (7)	−0.0262 (7)
F6	0.0314 (7)	0.0446 (8)	0.0286 (7)	0.0055 (6)	−0.0079 (5)	0.0077 (6)
O1	0.0407 (9)	0.0254 (8)	0.0406 (9)	−0.0040 (7)	−0.0226 (7)	0.0138 (7)
O2	0.0181 (6)	0.0113 (6)	0.0186 (7)	−0.0010 (5)	0.0008 (5)	−0.0002 (5)
O3	0.0142 (6)	0.0135 (6)	0.0274 (7)	−0.0029 (5)	0.0030 (5)	−0.0051 (5)
O4	0.0263 (7)	0.0178 (6)	0.0270 (7)	−0.0073 (5)	0.0003 (6)	−0.0023 (5)
O5	0.0304 (7)	0.0149 (6)	0.0204 (7)	0.0015 (5)	0.0002 (6)	−0.0003 (5)
O6	0.0361 (8)	0.0229 (7)	0.0206 (7)	−0.0018 (6)	0.0047 (6)	−0.0045 (6)
O7	0.0201 (7)	0.0249 (7)	0.0307 (8)	0.0029 (6)	0.0003 (6)	−0.0092 (6)
O8	0.0158 (7)	0.0213 (7)	0.0428 (9)	−0.0010 (5)	−0.0103 (6)	0.0069 (6)
O9	0.0161 (7)	0.0361 (8)	0.0410 (9)	−0.0091 (6)	−0.0044 (6)	0.0166 (7)
N1	0.0195 (8)	0.0156 (8)	0.0274 (9)	−0.0028 (6)	−0.0098 (7)	0.0008 (6)
C1	0.0215 (9)	0.0137 (8)	0.0227 (10)	−0.0035 (7)	0.0014 (7)	0.0017 (7)
C2	0.0166 (9)	0.0159 (9)	0.0296 (10)	−0.0072 (7)	0.0017 (8)	−0.0034 (7)
C3	0.0101 (8)	0.0140 (8)	0.0213 (9)	−0.0039 (6)	−0.0013 (7)	0.0010 (7)
C4	0.0094 (8)	0.0140 (8)	0.0230 (9)	−0.0024 (6)	−0.0024 (7)	−0.0004 (7)
C5	0.0110 (8)	0.0133 (8)	0.0200 (9)	−0.0022 (6)	−0.0047 (7)	0.0004 (7)
C6	0.0102 (8)	0.0129 (8)	0.0200 (9)	−0.0041 (6)	−0.0038 (7)	−0.0017 (7)
C7	0.0104 (8)	0.0111 (8)	0.0201 (9)	−0.0036 (6)	−0.0032 (7)	−0.0002 (6)
C8	0.0215 (10)	0.0214 (9)	0.0221 (10)	−0.0016 (7)	0.0037 (8)	0.0047 (8)
C9	0.0159 (9)	0.0185 (9)	0.0264 (10)	0.0026 (7)	0.0004 (8)	−0.0010 (7)
C10	0.0182 (9)	0.0161 (8)	0.0237 (10)	0.0010 (7)	−0.0052 (7)	0.0035 (7)
C11	0.0212 (9)	0.0168 (8)	0.0183 (9)	−0.0010 (7)	−0.0022 (7)	−0.0009 (7)
C12	0.0163 (9)	0.0115 (8)	0.0252 (10)	−0.0008 (7)	−0.0022 (7)	0.0018 (7)
C13	0.0199 (9)	0.0226 (9)	0.0283 (11)	0.0005 (8)	0.0001 (8)	−0.0047 (8)
C14	0.0261 (10)	0.0161 (9)	0.0255 (10)	−0.0049 (7)	−0.0031 (8)	−0.0022 (7)

### Geometric parameters (Å, °)

**Table:**

Ru1—N1	1.7817 (16)	C1—H1B	0.9900
Ru1—O3	2.1139 (13)	C2—H2A	0.9900
Ru1—O2	2.1252 (12)	C2—H2B	0.9900
Ru1—C5	2.1961 (17)	C3—C4	1.425 (2)
Ru1—C4	2.2008 (17)	C3—C7	1.444 (2)
Ru1—C7	2.2201 (16)	C3—C8	1.492 (3)
Ru1—C3	2.2247 (17)	C4—C5	1.445 (3)
Ru1—C6	2.2257 (17)	C4—C9	1.494 (2)
S1—O8	1.4328 (14)	C5—C6	1.434 (2)
S1—O9	1.4379 (14)	C5—C10	1.497 (2)
S1—O7	1.4633 (14)	C6—C7	1.425 (2)
S1—C13	1.818 (2)	C6—C11	1.491 (3)
S2—O6	1.4364 (14)	C7—C12	1.493 (2)
S2—O4	1.4383 (13)	C8—H8A	0.9800
S2—O5	1.4601 (14)	C8—H8B	0.9800
S2—C14	1.825 (2)	C8—H8C	0.9800
F1—C14	1.336 (2)	C9—H9A	0.9800
F2—C14	1.332 (2)	C9—H9B	0.9800
F3—C14	1.326 (2)	C9—H9C	0.9800
F4—C13	1.333 (2)	C10—H10A	0.9800
F5—C13	1.327 (2)	C10—H10B	0.9800
F6—C13	1.332 (2)	C10—H10C	0.9800
O1—N1	1.158 (2)	C11—H11A	0.9800
O2—C1	1.448 (2)	C11—H11B	0.9800
O2—H2	0.80 (3)	C11—H11C	0.9800
O3—C2	1.455 (2)	C12—H12A	0.9800
O3—H3	0.80 (3)	C12—H12B	0.9800
C1—C2	1.497 (3)	C12—H12C	0.9800
C1—H1A	0.9900		
			
N1—Ru1—O3	101.46 (7)	C3—C4—C9	125.28 (17)
N1—Ru1—O2	108.46 (6)	C5—C4—C9	126.77 (16)
O3—Ru1—O2	75.58 (5)	C3—C4—Ru1	72.14 (10)
N1—Ru1—C5	150.97 (7)	C5—C4—Ru1	70.63 (9)
O3—Ru1—C5	103.33 (6)	C9—C4—Ru1	124.63 (12)
O2—Ru1—C5	92.24 (6)	C6—C5—C4	107.92 (15)
N1—Ru1—C4	118.73 (7)	C6—C5—C10	126.86 (16)
O3—Ru1—C4	139.81 (6)	C4—C5—C10	125.12 (16)
O2—Ru1—C4	91.23 (6)	C6—C5—Ru1	72.20 (9)
C5—Ru1—C4	38.38 (7)	C4—C5—Ru1	70.99 (9)
N1—Ru1—C7	91.78 (7)	C10—C5—Ru1	125.25 (12)
O3—Ru1—C7	118.17 (6)	C7—C6—C5	107.99 (15)
O2—Ru1—C7	153.32 (6)	C7—C6—C11	126.16 (16)
C5—Ru1—C7	63.16 (6)	C5—C6—C11	125.82 (16)
C4—Ru1—C7	63.29 (6)	C7—C6—Ru1	71.09 (10)
N1—Ru1—C3	88.01 (7)	C5—C6—Ru1	69.95 (9)
O3—Ru1—C3	155.28 (6)	C11—C6—Ru1	123.16 (12)
O2—Ru1—C3	123.30 (6)	C6—C7—C3	108.26 (15)
C5—Ru1—C3	63.32 (6)	C6—C7—C12	126.20 (16)
C4—Ru1—C3	37.55 (6)	C3—C7—C12	125.11 (16)
C7—Ru1—C3	37.93 (6)	C6—C7—Ru1	71.52 (9)
N1—Ru1—C6	125.96 (7)	C3—C7—Ru1	71.21 (9)
O3—Ru1—C6	93.46 (6)	C12—C7—Ru1	128.90 (12)
O2—Ru1—C6	125.57 (6)	C3—C8—H8A	109.5
C5—Ru1—C6	37.85 (6)	C3—C8—H8B	109.5
C4—Ru1—C6	63.48 (6)	H8A—C8—H8B	109.5
C7—Ru1—C6	37.38 (6)	C3—C8—H8C	109.5
C3—Ru1—C6	62.99 (7)	H8A—C8—H8C	109.5
O8—S1—O9	116.77 (9)	H8B—C8—H8C	109.5
O8—S1—O7	113.43 (8)	C4—C9—H9A	109.5
O9—S1—O7	114.13 (9)	C4—C9—H9B	109.5
O8—S1—C13	104.41 (9)	H9A—C9—H9B	109.5
O9—S1—C13	103.72 (9)	C4—C9—H9C	109.5
O7—S1—C13	102.10 (9)	H9A—C9—H9C	109.5
O6—S2—O4	116.95 (9)	H9B—C9—H9C	109.5
O6—S2—O5	113.58 (8)	C5—C10—H10A	109.5
O4—S2—O5	113.84 (8)	C5—C10—H10B	109.5
O6—S2—C14	104.43 (9)	H10A—C10—H10B	109.5
O4—S2—C14	103.53 (9)	C5—C10—H10C	109.5
O5—S2—C14	102.23 (9)	H10A—C10—H10C	109.5
C1—O2—Ru1	115.58 (10)	H10B—C10—H10C	109.5
C1—O2—H2	110.5 (19)	C6—C11—H11A	109.5
Ru1—O2—H2	119.9 (19)	C6—C11—H11B	109.5
C2—O3—Ru1	112.50 (11)	H11A—C11—H11B	109.5
C2—O3—H3	106.7 (18)	C6—C11—H11C	109.5
Ru1—O3—H3	116.0 (18)	H11A—C11—H11C	109.5
O1—N1—Ru1	159.45 (14)	H11B—C11—H11C	109.5
O2—C1—C2	107.59 (14)	C7—C12—H12A	109.5
O2—C1—H1A	110.2	C7—C12—H12B	109.5
C2—C1—H1A	110.2	H12A—C12—H12B	109.5
O2—C1—H1B	110.2	C7—C12—H12C	109.5
C2—C1—H1B	110.2	H12A—C12—H12C	109.5
H1A—C1—H1B	108.5	H12B—C12—H12C	109.5
O3—C2—C1	105.25 (14)	F5—C13—F6	107.53 (16)
O3—C2—H2A	110.7	F5—C13—F4	107.46 (17)
C1—C2—H2A	110.7	F6—C13—F4	107.59 (17)
O3—C2—H2B	110.7	F5—C13—S1	111.59 (15)
C1—C2—H2B	110.7	F6—C13—S1	111.14 (14)
H2A—C2—H2B	108.8	F4—C13—S1	111.33 (14)
C4—C3—C7	107.89 (15)	F3—C14—F2	107.70 (16)
C4—C3—C8	125.55 (16)	F3—C14—F1	107.53 (16)
C7—C3—C8	126.49 (16)	F2—C14—F1	107.52 (16)
C4—C3—Ru1	70.32 (9)	F3—C14—S2	111.48 (14)
C7—C3—Ru1	70.86 (9)	F2—C14—S2	111.06 (13)
C8—C3—Ru1	126.86 (13)	F1—C14—S2	111.37 (13)
C3—C4—C5	107.92 (15)		
			
N1—Ru1—O2—C1	101.00 (13)	C6—Ru1—C5—C4	116.90 (14)
O3—Ru1—O2—C1	3.51 (11)	N1—Ru1—C5—C10	167.29 (15)
C5—Ru1—O2—C1	−99.68 (12)	O3—Ru1—C5—C10	−44.76 (16)
C4—Ru1—O2—C1	−138.07 (12)	O2—Ru1—C5—C10	30.94 (15)
C7—Ru1—O2—C1	−121.52 (15)	C4—Ru1—C5—C10	120.10 (19)
C3—Ru1—O2—C1	−158.89 (12)	C7—Ru1—C5—C10	−159.84 (18)
C6—Ru1—O2—C1	−80.33 (13)	C3—Ru1—C5—C10	157.47 (17)
N1—Ru1—O3—C2	−80.50 (13)	C6—Ru1—C5—C10	−123.0 (2)
O2—Ru1—O3—C2	25.85 (11)	C4—C5—C6—C7	−1.15 (18)
C5—Ru1—O3—C2	114.74 (12)	C10—C5—C6—C7	−177.64 (16)
C4—Ru1—O3—C2	100.18 (14)	Ru1—C5—C6—C7	61.24 (11)
C7—Ru1—O3—C2	−178.80 (11)	C4—C5—C6—C11	−179.44 (16)
C3—Ru1—O3—C2	168.68 (14)	C10—C5—C6—C11	4.1 (3)
C6—Ru1—O3—C2	151.73 (12)	Ru1—C5—C6—C11	−117.05 (17)
O3—Ru1—N1—O1	−137.2 (5)	C4—C5—C6—Ru1	−62.39 (11)
O2—Ru1—N1—O1	144.4 (5)	C10—C5—C6—Ru1	121.12 (17)
C5—Ru1—N1—O1	11.0 (6)	N1—Ru1—C6—C7	27.59 (13)
C4—Ru1—N1—O1	42.3 (5)	O3—Ru1—C6—C7	134.43 (10)
C7—Ru1—N1—O1	−17.9 (5)	O2—Ru1—C6—C7	−150.85 (9)
C3—Ru1—N1—O1	19.8 (5)	C5—Ru1—C6—C7	−118.20 (14)
C6—Ru1—N1—O1	−34.3 (5)	C4—Ru1—C6—C7	−79.96 (11)
Ru1—O2—C1—C2	−30.53 (18)	C3—Ru1—C6—C7	−37.71 (10)
Ru1—O3—C2—C1	−49.10 (17)	N1—Ru1—C6—C5	145.78 (11)
O2—C1—C2—O3	49.83 (19)	O3—Ru1—C6—C5	−107.38 (10)
N1—Ru1—C3—C4	146.55 (11)	O2—Ru1—C6—C5	−32.66 (12)
O3—Ru1—C3—C4	−99.89 (16)	C4—Ru1—C6—C5	38.23 (10)
O2—Ru1—C3—C4	35.68 (13)	C7—Ru1—C6—C5	118.20 (15)
C5—Ru1—C3—C4	−38.20 (10)	C3—Ru1—C6—C5	80.49 (11)
C7—Ru1—C3—C4	−118.00 (15)	N1—Ru1—C6—C11	−93.83 (15)
C6—Ru1—C3—C4	−80.83 (11)	O3—Ru1—C6—C11	13.01 (14)
N1—Ru1—C3—C7	−95.46 (11)	O2—Ru1—C6—C11	87.72 (15)
O3—Ru1—C3—C7	18.11 (19)	C5—Ru1—C6—C11	120.38 (18)
O2—Ru1—C3—C7	153.68 (9)	C4—Ru1—C6—C11	158.62 (16)
C5—Ru1—C3—C7	79.80 (11)	C7—Ru1—C6—C11	−121.42 (18)
C4—Ru1—C3—C7	118.00 (15)	C3—Ru1—C6—C11	−159.13 (16)
C6—Ru1—C3—C7	37.17 (10)	C5—C6—C7—C3	1.59 (18)
N1—Ru1—C3—C8	26.31 (16)	C11—C6—C7—C3	179.87 (16)
O3—Ru1—C3—C8	139.88 (15)	Ru1—C6—C7—C3	62.10 (11)
O2—Ru1—C3—C8	−84.55 (16)	C5—C6—C7—C12	174.32 (16)
C5—Ru1—C3—C8	−158.44 (17)	C11—C6—C7—C12	−7.4 (3)
C4—Ru1—C3—C8	−120.2 (2)	Ru1—C6—C7—C12	−125.16 (17)
C7—Ru1—C3—C8	121.77 (19)	C5—C6—C7—Ru1	−60.52 (11)
C6—Ru1—C3—C8	158.94 (17)	C11—C6—C7—Ru1	117.77 (17)
C7—C3—C4—C5	0.71 (19)	C4—C3—C7—C6	−1.43 (19)
C8—C3—C4—C5	−176.24 (16)	C8—C3—C7—C6	175.48 (16)
Ru1—C3—C4—C5	61.94 (11)	Ru1—C3—C7—C6	−62.30 (11)
C7—C3—C4—C9	178.56 (16)	C4—C3—C7—C12	−174.26 (16)
C8—C3—C4—C9	1.6 (3)	C8—C3—C7—C12	2.6 (3)
Ru1—C3—C4—C9	−120.22 (17)	Ru1—C3—C7—C12	124.86 (17)
C7—C3—C4—Ru1	−61.23 (11)	C4—C3—C7—Ru1	60.88 (11)
C8—C3—C4—Ru1	121.83 (17)	C8—C3—C7—Ru1	−122.21 (18)
N1—Ru1—C4—C3	−38.92 (13)	N1—Ru1—C7—C6	−157.97 (11)
O3—Ru1—C4—C3	140.32 (10)	O3—Ru1—C7—C6	−53.96 (11)
O2—Ru1—C4—C3	−150.82 (11)	O2—Ru1—C7—C6	61.92 (17)
C5—Ru1—C4—C3	117.13 (14)	C5—Ru1—C7—C6	37.30 (10)
C7—Ru1—C4—C3	37.41 (10)	C4—Ru1—C7—C6	80.51 (11)
C6—Ru1—C4—C3	79.42 (11)	C3—Ru1—C7—C6	117.56 (14)
N1—Ru1—C4—C5	−156.05 (10)	N1—Ru1—C7—C3	84.47 (11)
O3—Ru1—C4—C5	23.19 (14)	O3—Ru1—C7—C3	−171.52 (9)
O2—Ru1—C4—C5	92.06 (10)	O2—Ru1—C7—C3	−55.63 (17)
C7—Ru1—C4—C5	−79.71 (11)	C5—Ru1—C7—C3	−80.25 (11)
C3—Ru1—C4—C5	−117.13 (14)	C4—Ru1—C7—C3	−37.04 (10)
C6—Ru1—C4—C5	−37.70 (10)	C6—Ru1—C7—C3	−117.56 (14)
N1—Ru1—C4—C9	82.06 (16)	N1—Ru1—C7—C12	−35.94 (17)
O3—Ru1—C4—C9	−98.70 (16)	O3—Ru1—C7—C12	68.07 (17)
O2—Ru1—C4—C9	−29.83 (15)	O2—Ru1—C7—C12	−176.04 (13)
C5—Ru1—C4—C9	−121.89 (19)	C5—Ru1—C7—C12	159.34 (18)
C7—Ru1—C4—C9	158.40 (18)	C4—Ru1—C7—C12	−157.45 (18)
C3—Ru1—C4—C9	121.0 (2)	C3—Ru1—C7—C12	−120.4 (2)
C6—Ru1—C4—C9	−159.59 (17)	C6—Ru1—C7—C12	122.0 (2)
C3—C4—C5—C6	0.26 (19)	O8—S1—C13—F5	56.05 (16)
C9—C4—C5—C6	−177.54 (16)	O9—S1—C13—F5	−66.73 (16)
Ru1—C4—C5—C6	63.17 (11)	O7—S1—C13—F5	174.41 (14)
C3—C4—C5—C10	176.83 (16)	O8—S1—C13—F6	176.07 (13)
C9—C4—C5—C10	−1.0 (3)	O9—S1—C13—F6	53.29 (16)
Ru1—C4—C5—C10	−120.26 (17)	O7—S1—C13—F6	−65.57 (15)
C3—C4—C5—Ru1	−62.91 (12)	O8—S1—C13—F4	−64.01 (15)
C9—C4—C5—Ru1	119.29 (17)	O9—S1—C13—F4	173.21 (14)
N1—Ru1—C5—C6	−69.71 (18)	O7—S1—C13—F4	54.35 (15)
O3—Ru1—C5—C6	78.24 (10)	O6—S2—C14—F3	67.93 (15)
O2—Ru1—C5—C6	153.94 (10)	O4—S2—C14—F3	−54.97 (15)
C4—Ru1—C5—C6	−116.90 (14)	O5—S2—C14—F3	−173.49 (13)
C7—Ru1—C5—C6	−36.85 (10)	O6—S2—C14—F2	−52.17 (16)
C3—Ru1—C5—C6	−79.53 (11)	O4—S2—C14—F2	−175.08 (14)
N1—Ru1—C5—C4	47.19 (18)	O5—S2—C14—F2	66.40 (15)
O3—Ru1—C5—C4	−164.86 (9)	O6—S2—C14—F1	−171.97 (13)
O2—Ru1—C5—C4	−89.15 (10)	O4—S2—C14—F1	65.12 (15)
C7—Ru1—C5—C4	80.06 (10)	O5—S2—C14—F1	−53.40 (14)
C3—Ru1—C5—C4	37.37 (10)		

### Hydrogen-bond geometry (Å, °)

**Table:**

*D*—H···*A*	*D*—H	H···*A*	*D*···*A*	*D*—H···*A*
O2—H2···O5	0.80 (3)	1.76 (3)	2.568 (2)	176 (3)
O3—H3···O7	0.80 (3)	1.76 (3)	2.554 (2)	169 (3)

## References

[bb1] Bruker (2004). *APEX2* and *SAINT-Plus* Bruker AXS Inc., Madison, Wisconsin, USA.

[bb2] Burns, R. M. & Hubbard, J. L. (1994). *J. Am. Chem. Soc.* **116**, 9514–9520.

[bb3] Hubbard, J. L. & McVicar, W. K. (1992). *Inorg. Chem.* **31**, 910–913.

[bb4] Pearsal, M., Gembicky, M., Dominiak, P., Larsen, A. & Coppens, P. (2007). *Acta Cryst.* E**63**, m2596. 10.1107/S1600536810011980 PMC2979091 21578980

[bb5] Sheldrick, G. M. (1997). *SHELXS97 *and *SHELXL97* University of Göttingen, Germany.

[bb6] Sheldrick, G. M. (2000). *SHELXTL* Version 6.10. Bruker AXS Inc., Madison, Wisconsin, USA.

[bb7] Svetlanova-Larsen, A., Zoch, C. R. & Hubbard, J. L. (1996). *Organometallics*, **15**, 3076–3087.

[bb8] Westrip, S. P. (2008). *publCIF* In preparation.

[bb9] Yang, K., Bott, S. G. & Richmond, M. G. (1995). *J. Chem. Crystallogr.* **25**, 283–290.

[bb10] Yang, K., Martin, J. A., Bott, S. G. & Richmond, M. G. (1997). *Inorg. Chim. Acta*, **254**, 19–27.

