# (Glycol-κ^2^ *O*,*O*′)nitros­yl(η^5^-penta­methyl­cyclo­penta­dien­yl)ruthenium(II) bis­(tri­fluoro­methane­sulfonate). Corrigendum

**DOI:** 10.1107/S1600536810011992

**Published:** 2010-04-14

**Authors:** Semeret Munie, Anna Larsen, Milan Gembicky

**Affiliations:** a Department of Chemistry, CNS 359, Ithaca College, Ithaca, NY 14850, USA; b Department of Chemistry, State University of New York at Buffalo, 732 NSC Complex, Buffalo, NY 14260, USA

## Abstract

The acknowledgement in the paper by Munie, Larsen & Gembicky [*Acta Cryst.* (2008), E**64**, m293] is extended and an omitted reference is added.

## Abstract 2

Corrigendum to *Acta Cryst.* (2008), E**64**, m293.

In the paper by Munie *et al.* (2008) ▶, the complete acknow­ledgement should read: "Support of this research *via* the PRF 44692.01-GB award by the American Chemical Society and the Cottrell College Award CC6755 from Research Corporation is gratefully acknowledged. The title compound was first described in the context of ALs PhD dissertation completed in 1996 under the supervision of Dr John L. Hubbard, whose contribution to this project is gratefully acknowledged."A reference for the PhD dissertation [Larsen (née Svet­lan­ova), 1996 ▶] is also added.

## References

[bb2] Larsen (née Svetlanova), A. (1996). PhD dissertation, Utah State University, USA.

[bb1] Munie, S., Larsen, A. & Gembicky, M. (2008). *Acta Cryst.* E**64**, m293. 10.1107/S1600536807067426 PMC2960154 21201270

